# Optimization of Ultrasonic Extraction of Phenolic Compounds from *Epimedium brevicornum* Maxim Using Response Surface Methodology and Evaluation of Its Antioxidant Activities In Vitro

**DOI:** 10.1155/2014/864654

**Published:** 2014-11-12

**Authors:** Yan Zhao, Yingying Hou, Guosheng Tang, Enbo Cai, Shuangli Liu, He Yang, Lianxue Zhang, Shijie Wang

**Affiliations:** ^1^College of Chinese Medicinal Materials, Jilin Agricultural University, No. 2888 Xincheng Street, Changchun, Jilin 130118, China; ^2^College of Traditional Chinese Medicine, Jilin Agricultural Science and Technology University, Jilin 132101, China

## Abstract

The ultrasound-assisted extraction of phenolic compounds from *Epimedium brevicornu* Maxim was modeled using response surface methodology. A Central Composite Design (CCD) was employed to optimize three extraction variables, including ethanol concentration (*X*_1_), extraction time (*X*_2_), and ratio of aqueous ethanol to raw material (*X*_3_), for the achievement of high extraction yield of the phenolic compounds. The optimized conditions are *X*_1_ of 50% (v/v), *X*_2_ of 27.5 min, and *X*_3_ of 250 mL/g. Under these conditions, the experimental yield is 4.29 ± 0.033% (*n* = 3). The antioxidant activity was evaluated using the DPPH assay and ferric-reducing antioxidant power (FRAP). And it indicates that the phenolic compounds from *Epimedium brevicornu* Maxim possess significant antioxidant activity. HPLC analysis reveals that the main phenolic compound in the extract product was identified as gallic acid, catechin (Cianidanol), p-hydroxybenzoic acid, vanillic acid, caffeic acid, ferulaic acid, rutin, benzoic acid, and quercetin.

## 1. Introduction

The Herba Epimedii (family Berberidaceae) is widespread in Asia, Europe, and the Middle and Far East. And it is a famous Chinese herbal medicine, made from the dried aerial parts of* Epimedium brevicornum* Maxim,* Epimedium sagittatum* (Sieb. et Zucc.) Maxim,* Epimedium pubescens* Maxim,* Epimedium wushanense* T. S. Ying, and* Epimedium koreanum* Nakai [1]. It has been commonly used in the treatment of cardiovascular diseases and other chronic illness (infertility, amnesia and asthenia, impotence, and senile functional diseases) in China for over 2000 years [2].

Free radicals, chemical reactions, and several redox reactions of various compounds may cause protein oxidation, DNA damage, and lipid peroxidation in living cells [3]. Polyphenols, a large class of plant secondary metabolites, are important effective constituents of many natural products from plants and can protect the human body from free radicals and retard the progress of many chronic diseases. Currently, polyphenols have attracted extensive attention due to their various biological and pharmacological activities, including antioxidant, antibacterial, and anticancer activities [4, 5].

Ultrasonic-assisted extraction (UAE) is one of the most inexpensive, simple, rapid, and efficient green extraction techniques compared with conventional extraction [6] and has been applied to extract bioactive compounds from different materials due to its high reproducibility at shorter time, simplified manipulation, significant reduction in solvent consumption, and temperature, in respect to other classic methods [7, 8]. Therefore, the ultrasound technology has been used in some industries, such as food industry, chemical industry, and material industry [9].

Response surface methodology (RSM), an effective statistical technique for modeling and optimization of complex processes, has been used increasingly to optimize processing parameters owing to more efficient and easier arrangement and interpretation of experiments compared to others [8, 10, 11]. The advantage of RSM is the reduced number of experimental trials needed to evaluate multiple parameters and their interactions. Therefore, it is widely used in optimizing the extraction parameters, such as polysaccharides [12], anthocyanins [13], phenolic compounds [14], and protein [15] from different materials.

In this work, the aims of the present study were to investigate the extraction variables (ethanol concentration, extraction time, and ratio of aqueous ethanol to raw material); optimize these variables values by RSM for the phenolic compounds recovery yield maximization; and investigate the relationship between phenolic content and antioxidant activity from* Epimedium brevicornum *Maxim Furthermore, the antioxidant activity of the phenolic compounds was evaluated by DPPH assay and FRAP.

## 2. Materials and Methods

### 2.1. Materials and Reagents

*Epimedium brevicornum *Maxim was collected from Jilin province (China) and verified by Professor Lianxue Zhang (Jilin Agriculture University, Jilin, China). Folin-Ciocalteu phenol reagent, sodium carbonate, 2,2-diphenyl-1-picrylhydrazyl (DPPH), gallic acid, catechin (Cianidanol), vanillic acid, p-hydroxybenzoic acid, caffeic acid, ferulaic acid, rutin, benzoic acid, quercetin, and vitamin C (Vc) were from Aladdin (Shanghai, China). Methanol (HPLC grade) was purchased from Honeywell Burdick *＆* Jackson (Ulsan, Korea). Ultra-high purified water used in this study was prepared in a Milli-Q Water Purification System (Millipore, Bedford, MA, USA). Other chemicals used were of analytical grade.

### 2.2. Extraction of Phenolic Compounds

The process of phenolic compounds extraction from* Epimedium brevicornum* Maxim by ultrasonic-assisted treatment was performed in an ultrasonic generator (250 W, 40 kHz, KQ-250B, Kunshan, China). The dry* Epimedium brevicornum* Maxim was powdered by a pulverizer (Fw-200, Beijing, China) and then passed through an 80 mesh sieve. One gram of the* Epimedium brevicornum* Maxim powders was used for each case in a beaker. The beaker was held in the ultrasonic generator and exposed to extract phenolic compounds for different extraction time at various ethanol concentrations in different ratios of aqueous ethanol to raw material.

### 2.3. Total Phenolic Content (TPC) Determination

After ultrasonic treatment, the extracted slurry was centrifuged at 4000 rpm for 15 min to collect the supernatant. The content of total phenols was determined by the Folin-Ciocalteu method [16]. Briefly, diluted sample (0.50 mL) was added to 1 : 10 diluted Folin-Ciocalteu reagent (2.5 mL). After 4 min, saturated sodium carbonate solution (about 75 g/L, 2 mL) was added. After 2 h of incubation at room temperature, the absorbance of the reaction mixture was measured at 760 nm. Gallic acid was used as a reference standard, and the results were expressed as milligram gallic acid equivalent (mg GAE)/g dry weight of plant material. All the experiments were performed in triplicate, and the results were expressed as mean ± SD (standard deviation). The correlation between the antioxidant capacities and total phenolic contents was analyzed using the simple linear regression, and the correlation coefficient (*R* ^2^) was calculated.

### 2.4. Determination of In Vitro Antioxidant Assays

#### 2.4.1. Reducing Ability Assay

The ferric-reducing antioxidant power (FRAP) was assessed according to a reported procedure with minor modifications [17]. Various concentrations of* Epimedium brevicornum* Maxim (5~1000 *μ*g/mL) in sodium phosphate buffer (1.5 mL, 0.2 M, pH = 6.6) were mixed with potassium ferricyanide (1.5 mL, 1%, w/v) and the mixture was incubated at 50°C for 20 min. After that, trichloroacetic acid (TCA, 1.5 mL, 10%, w/v) was added, and the mixture was centrifuged at 3,000 rpm for 15 min. The centrifugate (1.5 mL) was mixed thoroughly with deionized water (1.5 mL) and FeCl_3_ (0.3 mL, 0.1%, w/v), and the absorbance was measured at 700 nm. The increase in absorbance of the reaction mixture indicated reducing power. The reducing power was expressed as EC_50_ (*μ*g/mL), which is the concentration of the sample to cause a 0.5 OD at 700 nm. Butylated hydroxyl toluene (BHT) was used as standard.

#### 2.4.2. DPPH Radical Scavenging Assay

DPPH^•^ quenching ability of phenolic compoundsfrom* Epimedium brevicornum* Maxim was measured according to Hanato's method [18]. A methanol DPPH solution (0.15%) was mixed with serial dilutions (5~1000 *μ*g/mL) of* Epimedium brevicornum* Maxim and after 10 min, the absorbance was read at 515 nm. The antiradical activity was expressed as IC_50_ (*μ*g/mL), the antiradical dose required to cause a 50% inhibition. Vitamin C was used as standard. The ability to scavenge the DPPH radical was calculated using the following equation:

(1) $$\begin{array}{c} \text{DPPH}\;\;\text{scavenging}\;\;\text{effect}\;\left(\%\right)=\frac{\left(A_{0}-A_{1}\right)}{A_{0}}\times 100, \end{array}$$

where *A*_0_ is the absorbance of the control at 30 min and *A*_1_ is the absorbance of the sample at 30 min. All samples were analyzed in triplicate.

### 2.5. HPLC Analysis

The extract phenolics were analyzed by HPLC method [16] with some modifications. Using a HPLC (CXTH-3000 series, Beijing Tong Heng Innovation Technology Co., Ltd., China) equipped with a UV detector (LC3000, Beijing Tong Heng) and a C_18_ column (5 *μ*m, 250 mm × 4.6 mm, Dalian Jiangshen separating technology Co., Ltd.). Ultrapure water was used as solvent* A* and 100% methanol as solvent* B* (0~40 min, *A*: 5%~60%; 40~50 min, *A*: 60%~60%). The solutions of the standards and the extract phenolics were filtered through a 0.45 nm syringe filter. The operating conditions were column temperature, 30°C; injection volume, 20 *μ*L; detection wavelength, 280 nm; flow rate, 1.0 mL/min. The identification and peak assignment of the phenolics were based on comparison of retention times and spectral data with those of the standards. The identified phenolics were quantified according to respective standard calibration curves.

### 2.6. Experimental Design

A Central Composite Design (CCD) was employed to determine the best combination of extraction variables for the phenolic compounds based on the results of preliminary single-factor-test. Extraction time (*X*_1_), ethanol concentration (*X*_2_), and ratio of aqueous ethanol to raw material (*X*_3_) were the independent variables, and their uncoded and coded levels were presented in Table 1. Extraction yield (*Y*) taken as the response for the design experiment was given in Table S1, Table S2, Table S3, Figure S1a, Figure S1b, Figure S1c, Figure S2, Figure S3, Figure S4, were displayed in Supplementary Material which would be available online at http://dx.doi.org/10.1155/2014/864654.

### 2.7. Statistical Analysis

All the data were determined in triplicate and the results were averaged. SPSS software version 18 was used to evaluate the DPPH assay and FRAP. Design Expert software version 8.0.6.1 (Stat-Ease, Minneapolis) was employed for the regression analysis and the optimization.

## 3. Results and Discussion

### 3.1. Effect of Ethanol Concentration on Extraction Yield of Phenolic Compounds

Extraction process was carried out at different ethanol concentrations of 5%, 10%, 20%, 40%, 60%, 80%, and 95%, while other parameters were as follows: extraction time 30 min and ratio of aqueous ethanol to raw material 300 mL/g. The effect of ethanol concentration on extraction yield of phenolic compounds is shown in Figure S1a. The extraction yield was calculated using the following equation:

(2) $$\begin{array}{c} \text{Extraction}\;\;\text{yield}\;\left(\%\right)=\frac{A_{0}}{A_{1}}\times 100, \end{array}$$

where *A*_0_ is the content of total phenolic compounds and *A*_1_ is the quality of* Epimedium brevicornum *Maxim. All samples were analyzed in triplicate.

The variance of extraction yield increases first, then decreases with the increase of ethanol concentration, and peaks at 60% (v/v). Ethanol/water was chosen as the unique extraction solvent, instead of others, because of low price of ethanol, low toxicity, easiness of recycling (which is good from an environmental point of view), and good polarity to extract the components of interest. It is reported that water is acting as the plant swelling agent, while ethanol is believed to disrupt the bonding between the solutes and plant matrices [19]. Moreover, water has a high dielectric constant, which leads to different ethanol concentrations with different polarities [20]. Therefore, the results may be related to the solvent polarity and the solubility of polyphenols in* Epimedium brevicornum *Maxim, and the ethanol concentration of 60% (v/v) is good for extracting the phenolic compounds.

### 3.2. Effect of Extraction Time on Extraction Yield of Phenolic Compounds

Extraction process was carried out using extraction time from 5 to 50 min, while other parameters were as follows: ethanol concentration 60% (v/v) and ratio of aqueous ethanol to raw material 300 mL/g. The effect of extraction time on extraction yield of phenolic compounds from* Epimedium brevicornum *Maxim is shown in Figure S1b. When extraction time increases, the variance of extraction yield is relatively rapid and reaches a maximum at 40 min and then decreases as the extraction proceeds, possibly due to the structural destruction and the decomposition of polyphenols during the prolonged extraction time [21]. Therefore, 40 min is favorable for extracting the phenolic compounds.

### 3.3. Effect of Ratio of Aqueous Ethanol to Raw Material on Extraction Yield of Phenolic Compounds

Extraction process was carried out using ratio of aqueous ethanol to raw material in the range of 20 to 300 mL/g, while extraction time and ethanol concentration were fixed at 40 min and 60% (v/v), respectively. The effect of ratio of aqueous ethanol to raw material on extraction yield of phenolic compounds is shown in Figure S1c. As ratio of aqueous ethanol to raw material increases, the extraction yield slowly increases first and a maximum yield is achieved at 250 mL/g, and then slightly decreases after the ratio of aqueous ethanol to raw material exceeds 250 mL/g. This phenomenon may be attributed to the mass transfer principle and the distribution of ultrasonic energy density in the extraction solutions [22]. Lower ratio of aqueous ethanol to raw material has higher concentration gradient, leading to higher diffusion and extraction yield. But when the ratio is over 250 mL/g, the decrease of the distribution of ultrasonic energy density in the extraction solutions is dominant and has a negative effect on the extraction yield. Therefore, the ratio of aqueous ethanol to raw material of 250 mL/g is sufficient for extracting the phenolic compounds.

### 3.4. Optimization of Extraction Parameters for Phenolic Compounds

Table S1 shows the process variables and experimental data of 17 runs containing 3 replicates at center point. By applying multiple regression analysis on the experimental data, the model for the response variable could be expressed by the following quadratic polynomial equation in the form of coded values:

(3) Y=−3.72212+0.11831∗X1 +0.11269∗X2+0.03147∗X3 −0.00021∗X1∗X1 +0.00005∗X1∗X2 +0.00004∗X2∗X2 −0.00126∗X1∗X3 −0.00165∗X2∗X3 −0.00008∗X3∗X3.

Analysis of variance (ANOVA) for the model is shown in Table S2. The determination coefficient (*R* ^2^ = 0.9905) indicates that only 0.95% of the total variations are not explained by the model. For a good statistical model, the adjusted determination coefficient (*R* ^2^ adj) should be close to *R* ^2^. As shown in Table S2,  *R* ^2^ adj (0.9782) is close to *R* ^2^. Moreover, *R* ^2^ pred (0.9664) is in reasonable agreement with *R* ^2^ adj and confirms that the model is highly significant. The lack of fit test determines whether the selected model is adequate to explain the experimental data, or whether another model should be reselected. The value of lack of fit test (0.9720) is higher than 0.05, which is not significant relative to the pure error and indicates that the fitting model is adequate to describe the experimental data. An adequate precision is a measure of the signal to noise ratio, and the ratio of signal to noise is greater than 4 considered to be desirable [23]. The value of adequate precision is 26.402, demonstrating an adequate signal. At the same time, a relatively low value of coefficient of variation (CV) (3.25) indicates a better precision and reliability of the experimental values. Therefore, the model is adequate for prediction in the range of experimental variables.

The significance of each coefficient measured using *P* value and *F* value is listed in Table S3. Smaller *P* value and greater *F* value mean the corresponding variables would be more significant. The *P* value of the model is less than 0.0001, which indicates that the model is significant and can be used to optimize the extraction variables. The three independent variables (*X*_1_, *X*_2_, and *X*_3_) and three quadratic terms (*X*_1_ *X*_3_, *X*_2_ *X*_3_, and *X*_3_ *X*_3_) significantly affect the extraction yield within a 99% confidence interval, and the interaction between extraction time (*X*_1_) and ethanol concentration (*X*_2_), as well as extraction time (*X*1) and extraction time (*X*_1_), is significant (*P* < 0.01). Meanwhile, ratio of aqueous ethanol to raw material (*X*_3_) is the most significant factor affecting the extraction yield.

### 3.5. Analysis of Response Surfaces

2D contour plots and 3D response surface are the graphical representations of regression equation and are very useful to judge the relationship between independent and dependent variables. Different shapes of the contour plots indicate whether the mutual interactions between the variables are significant or not. Circular contour plot means the interactions between the corresponding variables are negligible, while elliptical contour suggests the interactions between the corresponding variables are significant. The three-dimensional representation of the response surfaces and two-dimensional contours generated by the model are shown in Figures S2–S4. In these three variables, when two variables are depicted in three-dimensional surface plots, the third variable is fixed at zero level. It is found in Figures S2–S4 that all the three response surfaces are convex in shape, which indicates that the ranges of variables were chosen properly. As shown in Figure S2, extraction yield increases rapidly when ethanol concentration (*X*_1_) and extraction time (*X*_2_) increase in the range of 20–47.8% (v/v) and 5–32.5 min, respectively; but beyond 47.8% and 32.5 min, extraction yield decreases slightly. This demonstrates that the effect of ethanol concentration (*X*_1_) and extraction time (*X*_2_) on extraction yield is significant and is in good agreement with the results in Table S3. Moreover, the elliptical contour plots in Figure S2 mean that there is a significant interaction between the two variables, which also agrees with the results in Table S3. It is obvious in Figure S3; both ethanol concentration (*X*_1_) and ratio of aqueous ethanol to raw material (*X*_3_) have quadratic effect on extraction yield. Extraction yield increases at first and then decreased quickly with increasing of the two parameters, and a maximum extraction yield is achieved when ethanol concentration (*X*_1_) and ratio of aqueous ethanol to raw material (*X*_3_) are 49.5% (v/v) and 219.0 mL/g, respectively. It can be seen that the mutual interactions between ethanol concentration (*X*_1_) and ratio of aqueous ethanol to raw material (*X*_3_) are significant due to the elliptical contour plots shown in Figure S3, which is also confirmed by the results in Table S3. From Figure S4, both extraction time (*X*_2_) and ratio of aqueous ethanol to raw material (*X*_3_) have quadratic effect on extraction yield. Extraction yield increases at first and then decreased quickly with increasing of the two parameters, and a maximum extraction yield is achieved when extraction time (*X*_2_) and ratio of aqueous ethanol to raw material (*X*_3_) are 34.5 min and 220.0 mL/g, respectively. It can be seen that the mutual interactions between extraction time (*X*_2_) and ratio of aqueous ethanol to raw material (*X*_3_) are significant due to the elliptical contour plots shown in Figure S4, which is also confirmed by the results in Table S3.

### 3.6. Verification of the Model

The suitability of the model equation for predicting the optimum response values is tested using the selected optimum conditions. The optimum conditions are ethanol concentration (*X*_1_) of 50.8% (v/v), extraction time (*X*_2_) of 27.9 min, and ratio of aqueous ethanol to raw material (*X*_3_) of 250.7 mL/g, under which the predicted yield is 4.28%. However, considering the operability in actual production, the optimum conditions are modified as follows: ethanol concentration (*X*_1_) of 50% (v/v), extraction time (*X*_2_) of 28 min, and ratio of aqueous ethanol to raw material (*X*_3_) of 250 mL/g, under which the experimental yield is 4.29 ± 0.033% (*n* = 3), agreeing closely with the predicted yield and consequently indicating the RSM model is satisfactory and accurate.

### 3.7. Reducing Power of* Epimedium brevicornum* Maxim

**Figure 1:**
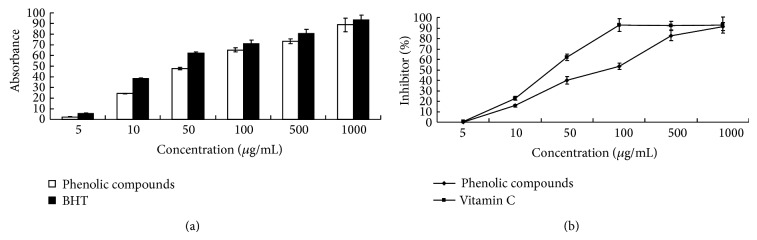
(a) Reductive ability of EFP and BHT. (b) DPPH radical scavenging effect of EFP and vitamin C.

The results of* Epimedium brevicornum *Maxim reducing power were showed in Figure 1(a). It shows the reductive capabilities of* Epimedium brevicornum *Maxim compared to the standard BHT; the reducing power of* Epimedium brevicornum *Maxim increased with increasing quantity of the sample. The EC_50_ value of* Epimedium brevicornum *Maxim and BHT was 69.2 ± 2.44 *μ*g/mL and 35.7 ± 0.81 *μ*g/mL, respectively. These results indicate that the phenolic compounds from* Epimedium brevicornum *Maxim showed very high reducing power in vitro.

### 3.8. Scavenging Activity on DPPH Radical

DPPH, a stable nitrogen centered free radical, has been used to evaluate natural antioxidants for their radical quenching capacities in a relatively short time, compared with other methods [24]. The scavenging activities of* Epimedium brevicornum *Maxim on DPPH free radical compared to the standard vitamin C were shown in Figure 1(b).* Epimedium brevicornum *Maxim exhibited a significant dose dependent inhibition of DPPH activity, with a 50% inhibition (IC_50_) at a concentration of 86.40 ± 0.62 *μ*g/mL. The IC_50_ value of vitamin C was 33.59 ± 0.31 *μ*g/mL. These results indicate that the phenolic compounds from* Epimedium brevicornum *Maxim have a noticeable effect on scavenging DPPH free radicals.

### 3.9. The Correlation between Antioxidant Activity and Total Phenolic Content

The correlation coefficient (*R* ^2^) between the antioxidant activity and the total phenolic content of the* Epimedium brevicornum* Maxim was determined (Table 2). The antioxidant activity and the total phenolic content showed a good correlation in both the FRAP (*R* ^2^ = 0.793) and DPPH (*R* ^2^ = 0.913) extracts. And the FRAP also showed a good correlation in DPPH (*R* ^2^ = 0.812).

### 3.10. HPLC Analysis of Extract Composition and Polyphenol Content

**Figure 2:**
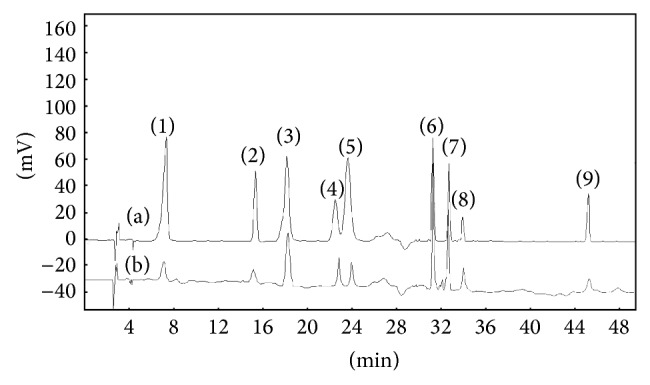
HPLC chromatograms. (a) HPLC chromatograms of a standard solution ((1) gallic acid = 20 *μ*g/mL, (2) catechin = 20 *μ*g/mL, (3) p-hydroxybenzoic acid = 20 *μ*g/mL, (4) vanillic acid = 22 *μ*g/mL, (5) caffeic acid = 20 *μ*g/mL, (6) ferulaic acid = 19 *μ*g/mL, (7) rutin = 20 *μ*g/mL, (8) benzoic acid = 25 *μ*g/mL, (9) quercetin = 10 *μ*g/mL). (b) Typical HPLC chromatogram obtained by direct injection of 20 *μ*L of extract of polyphenols from* Epimedium brevicornum *Maxim by optimized conditions (ethanol concentration 50%, extraction time 27.5 min, and ratio of aqueous ethanol to raw material 250 mL/g).

Figure 2 shows the chromatograms of the standard mixture and the extracts. The HPLC chromatograms reveal that gallic acid, catechin, p-hydroxybenzoic acid, vanillic acid, caffeic acid, ferulaic acid, rutin, benzoic acid, and quercetin are the major phenolic compounds in* Epimedium brevicornum* Maxim. The content of gallic acid, catechin, p-hydroxybenzoic acid, vanillic acid, caffeic acid, ferulaic acid, rutin, benzoic acid, and quercetin in* Epimedium brevicornum* Maxim is calculated from respective standard calibration curves which is shown in Table 3 and the values are 0.14% for gallic acid, 1.50% for catechin, 0.50% for p-hydroxybenzoic acid, 0.13% for vanillic acid, 0.07% for caffeic acid, 0.05% for ferulaic acid, 1.12% for rutin, 0.19% for benzoic acid, and 0.14% for quercetin. These results indicate that catechin and rutin may be mainly responsible for the antioxidant activity.

## 4. Conclusions

In the present study, the conditions for enhanced extraction of polyphenols from* Epimedium brevicornum *Maxim by UAE were optimized with a Central Composite Design based on response surface methodology.

Based on the single-factor-test, Central Composite Design was used to evaluate and ptimize the extraction variables (ethanol concentration, extraction time, and ratio of aqueous ethanol to raw material) for the extraction yield. The optimized conditions are as follows: ethanol concentration 50%, extraction time 27.5 min, and ratio of aqueous ethanol to raw material 250 mL/g. Under these conditions, the experimental yield is 4.29%, which agreed closely with the predicted yield of 4.28%. This optimized extraction has increased the total phenolic content yield significantly by 1.68% when compared to previous studies conducted by Wong et al. [25]. The antioxidant activity was evaluated using the DPPH assay and ferric-reducing antioxidant power (FRAP). And it indicates that the phenolic compounds from* Epimedium brevicornum *Maxim possess significant antioxidant activity. The HPLC analysis and the DPPH assay indicate that the extracts are composed of gallic acid, catechin, p-hydroxybenzoic acid, vanillic acid, caffeic acid, ferulaic acid, rutin, benzoic acid, and quercetin and have significant antioxidant activity.

## Supplementary Material

The results of the single-factor-test and Central Composite Design tests.

## Figures and Tables

**Table 1:** Independent variables and their levels used in the response surface design.

Factor	Notation	level				
		−1.682	−1	0	1	1.682
Ethanol concentration (%) (v/v)	*X*_1_	20	32.16	50	67.84	80
Ultrasonic extraction time (min)	*X*_2_	5	14.12	27.5	40.88	50
Liquid-solid ratio (mL/g)	*X*_3_	50	90.55	150	209.45	250

**Table 2:** Correlation between total phenolic content (TPC), DPPH radical scavenging ability (DPPH), and ferric-reducing antioxidant power (FRAP).

	DPPH	FRAP	TPC
DPPH		0.812	0.913
FRAP	0.812		0.793
TPC	0.913	0.793	

**Table 3:** Summary of HPLC method performance: linear equation, linear ranges, and coefficients of phenolic compounds (*n* = 3).

Phenolic compounds	Retention time (min)	Slope	Intercept	Linear range (*μ*mol/mL)	*R* ^2^
Gallic acid	7.23 ± 0.07	1.20 × 10^9^ ± 0.06 × 10^9^	−1.00 × 10^4^ ± 0.55 × 10^4^	0.01–1.18	0.999
Catechin	15.51 ± 0.11	1.18 × 10^8^ ± 0.04 × 10^8^	−5.17 × 10^2^ ± 0.14 × 10^2^	0.01–0.65	0.999
p-Hydroxybenzoic acid	17.61 ± 0.11	5.78 × 10^8^ ± 0.12 × 10^8^	2.96 × 10^3^ ± 1.13 × 10^3^	0.01–1.45	0.999
Vanillic acid	22.92 ± 0.12	7.34 × 10^8^ ± 0.23 × 10^8^	−6.89 × 10^3^ ± 1.66 × 10^3^	0.01–1.31	0.999
Caffeic acid	23.94 ± 0.12	1.24 × 10^9^ ± 0.07 × 10^9^	−1.50 × 10^4^ ± 0.6 × 10^4^	0.01–1.11	0.999
Ferulaic acid	31.73 ± 0.09	8.15 × 10^9^ ± 0.25 × 10^9^	−4.15 × 10^3^ ± 1.66 × 10^3^	0.01–0.20	0.999
Rutin	32.34 ± 0.13	8.26 × 10^8^ ± 0.49 × 10^8^	1.99 × 10^3^ ± 0.40 × 10^3^	0.01–0.33	0.999
Benzoic acid	34.11 ± 0.14	9.55 × 10^7^ ± 0.36 × 10^7^	3.32 × 10^3^ ± 0.98 × 10^3^	0.02–2.05	0.999
Quercetin	45.49 ± 0.15	9.61 × 10^8^ ± 0.33 × 10^8^	−5.11 × 10^3^ ± 1.23 × 10^3^	0.01–0.33	0.999
