# Supplementary material for: Optimization of Ultrasonic Extraction of Phenolic Compounds from Epimedium brevicornum Maxim Using Response Surface Methodology and Evaluation of Its Antioxidant Activities In Vitro
Source: J Anal Methods Chem. 2014 Nov 12;2014:864654. doi: 10.1155/2014/864654 (PMC4244914; doi:10.1155/2014/864654)
Supplement: Supplementary file 1 — The results of the single-factor-test and Central Composite Design tests. [file 864654.f1.pdf]

Fig. S1. Effect of different extraction variables on extraction yield.

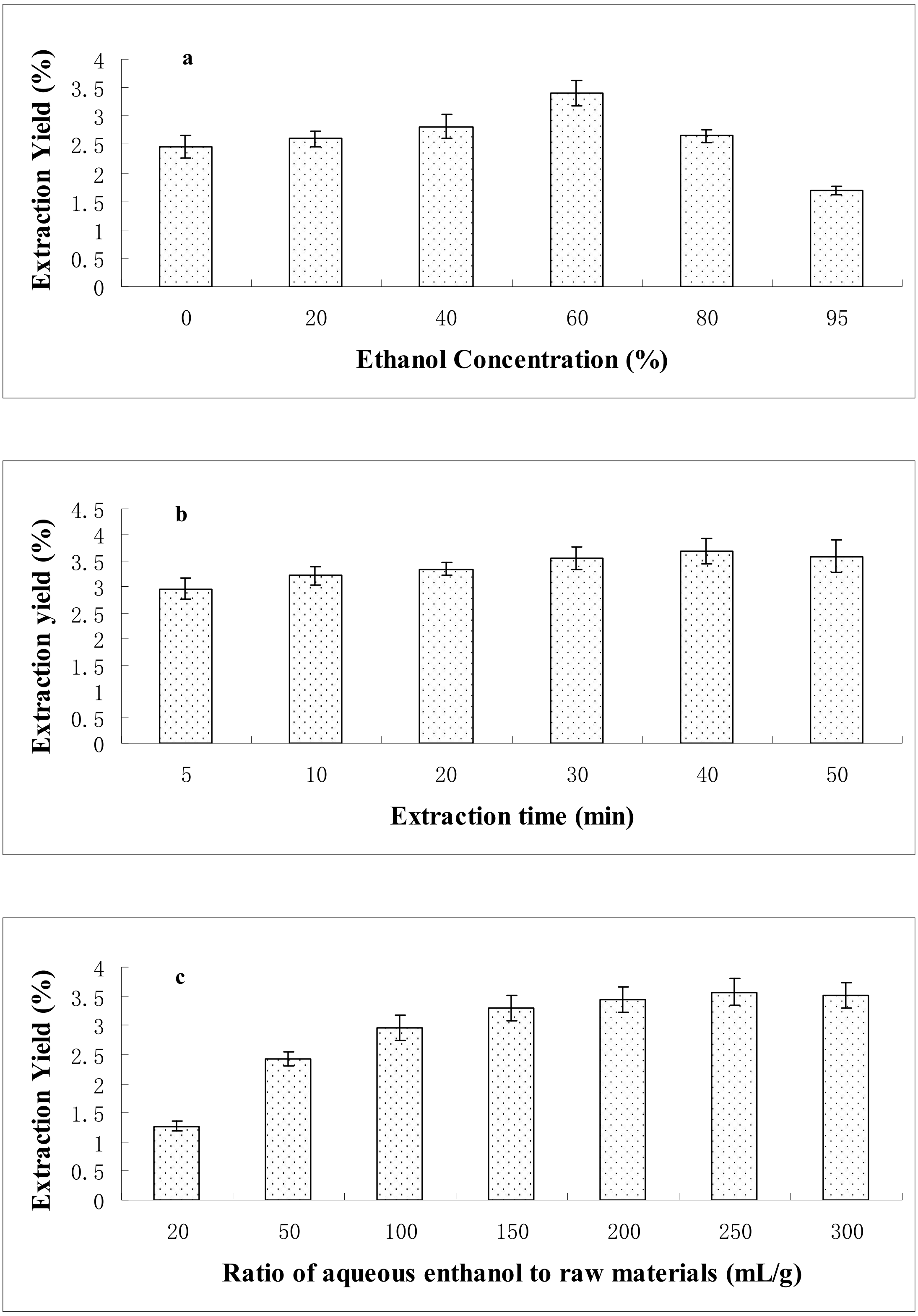

Fig. S2. Response surface and contour plots showing effect of ethanol concentration ($X_1$) and extraction time ($X_2$).

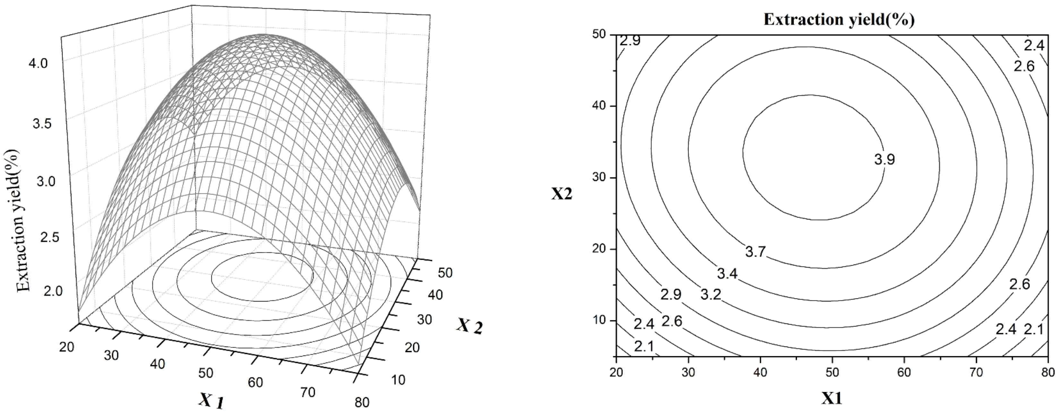

Fig. S3. Response surface and contour plots showing effect of ethanol concentration ($X_1$) and ratio of aqueous ethanol to raw material ($X_3$).

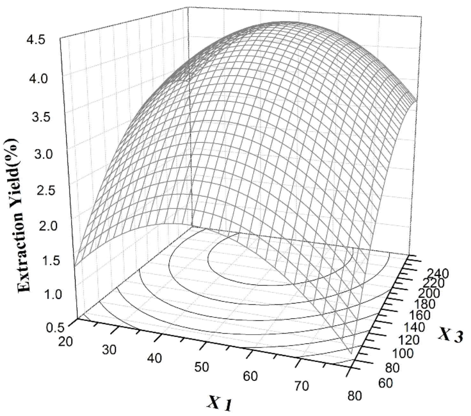


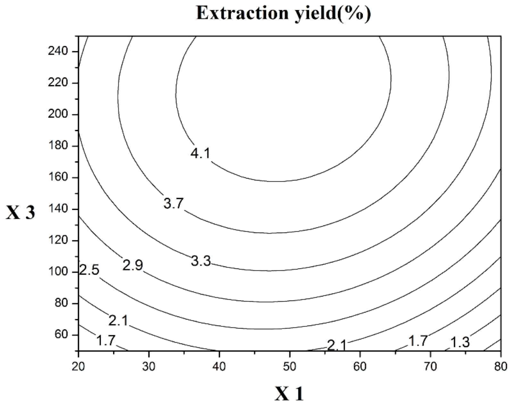

Fig. S4. Response surface and contour plots showing effect of extraction time ($X_2$) and ratio of aqueous ethanol to raw material ($X_3$).

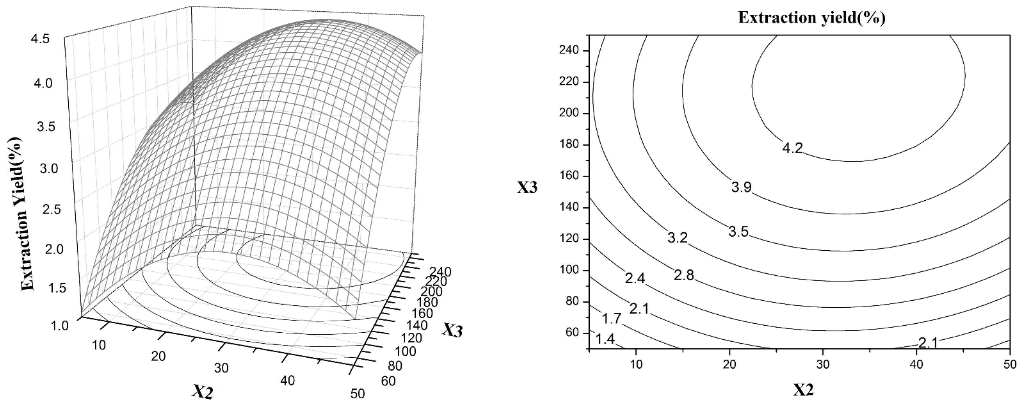

Table S1

Response surface central composite design and results for extraction yield of phenolic compounds.

| Run order | $x_1$ | $x_2$ | $x_3$ | Recovery (%) |
|---|---|---|---|---|
| 1 | 50（0） | 27.5（0） | 150（0） | 4.00 |
| 2 | 50（0） | 27.5（0） | 150（0） | 3.99 |
| 3 | 50（0） | 27.5（0） | 150（0） | 3.70 |
| 4 | 67.84（+1） | 40.88（+1） | 209.45（+1） | 3.83 |
| 5 | 67.84（+1） | 40.88（+1） | 90.55（-1） | 2.41 |
| 6 | 67.84（+1） | 14.12（-1） | 209.45（+1） | 3.44 |
| 7 | 67.84（+1） | 14.12（-1） | 90.55（-1） | 2.12 |
| 8 | 32.16（-1） | 40.88（+1） | 209.45（+1） | 4.02 |
| 9 | 32.16（-1） | 40.88（+1） | 90.55（-1） | 2.80 |
| 10 | 32.16（-1） | 14.12（-1） | 209.45（+1） | 3.41 |
| 11 | 32.16（-1） | 14.12（-1） | 90.55（-1） | 2.12 |
| 12 | 20（-1.682） | 27.5（0） | 150（0） | 3.08 |
| 13 | 80（+1.682） | 27.5（0） | 150（0） | 2.73 |
| 14 | 50（0） | 5（-1.682） | 150（0） | 3.02 |
| 15 | 50（0） | 50（+1.682） | 150（0） | 3.59 |
| 16 | 50（0） | 27.5（0） | 50（-1.682） | 2.16 |
| 17 | 50（0） | 27.5（0） | 250（+1.682） | 4.29 |

Table S2

Analysis of variance for fitted quadratic model of extraction of phenolic compounds.

| Source | Sum of squares | Degree of freedom | Mean square | F-value | p-Value（Prob.>F) |
|---|---|---|---|---|---|
| Model | 8.01 | 9 | 0.89 | 80.80 | <0.0001 |
| Residual | 0.0077 | 7 | 0.011 | | |
| Lack of fit | 0.018 | 5 | 0.003689 | 0.13 | 0.9720 |
| pure error | 0.059 | 2 | 0.029 | | |
| Cor Total | 8.08 | 16 | | | |

$R^2$=0.9905; adj $R^2$=0.9782; Pred $R^2$=0.9664; C.V. %=3.25; Adeq Precision=26.402

Table S3

Regression coefficients estimate and their significance test for quadratic model.

| Source | Sum of Squares | Degree of freedom | Mean Square | F-Value | p-value (Prob > F) |
|---|---|---|---|---|---|
| x1 | 0.1352 | 1 | 0.1352 | 115.8987 | < 0.0001 |
| x2 | 0.6832 | 1 | 0.6832 | 585.6896 | < 0.0001 |
| x3 | 5.4483 | 1 | 5.4483 | 4670.9709 | < 0.0001 |
| x1*x1 | 0.0205 | 1 | 0.0205 | 17.5817 | 0.0041 |
| x2*x1 | 0.0250 | 1 | 0.0250 | 21.3953 | 0.0024 |
| x2*x2 | 0.0064 | 1 | 0.0064 | 5.4496 | 0.0523 |
| x3*x1 | 1.8109 | 1 | 1.8109 | 1552.5369 | < 0.0001 |
| x3*x2 | 0.9818 | 1 | 0.9818 | 841.6878 | < 0.0001 |
| x3*x3 | 0.9359 | 1 | 0.9359 | 802.3542 | < 0.0001 |
